# How I Treat: STAT3 hyper IgE syndrome

**DOI:** 10.70962/jhi.20260038

**Published:** 2026-05-29

**Authors:** Cliodhna Ella Murray, Eyad Jannoud, Seraina Prader, Bodo Grimbacher

**Affiliations:** 1 Clinic of Rheumatology and Clinical Immunology, Center for Chronic Immunodeficiency, Medical Center, Faculty of Medicine, Albert-Ludwigs-University of Freiburg, Freiburg im Breisgau, Germany; 2 Institute for Immunodeficiency, Center for Chronic Immunodeficiency, Medical Center, Faculty of Medicine, Albert-Ludwigs-University of Freiburg, Freiburg im Breisgau, Germany; 3 Division of Immunology, University Children’s Hospital Zurich, Zurich, Switzerland; 4 German Center for Infection Research, Satellite Center Freiburg, Germany; 5 Centre for Integrative Biological Signalling Studies, Albert-Ludwigs University, Freiburg, Germany; 6 RESIST – Cluster of Excellence (EXC 2155) to Hannover Medical School, Satellite Center Freiburg, Germany

## Abstract

STAT3 hyper IgE syndrome (STAT3-HIES) is a rare inborn error of immunity characterized by chronic eczema, recurrent bacterial and fungal infections, markedly elevated serum IgE levels, and a broad spectrum of skeletal, dental, vascular, and gastrointestinal manifestations. Despite increasing insight into the genetic and immunologic basis of STAT3-HIES, detailed, treatment-oriented guidance for clinical practice remains limited. This article provides a pragmatic management guideline for STAT3-HIES. Clinical and laboratory criteria that should prompt suspicion and select patients for genetic testing are discussed. The therapeutic sections cover acute and prophylactic antibacterial treatment, antimicrobial eradication strategies, antifungal therapy, and immunoglobulin replacement therapy. The treatment of severe eczema, organ-specific care for lung, skeletal, vascular, and dental involvement, vaccinations, and supportive measures including physiotherapy and psychosocial support, as well as approaches to pregnancy management, family planning, and genetic counseling are outlined. Furthermore, hematopoietic stem cell transplantation is discussed. This management-focused approach complements existing reviews and aims to standardize care for patients with STAT3-HIES across centers and disciplines.

## Teaser

The hyper IgE syndrome (formerly also called Job syndrome or Buckley syndrome) is a rare and syndromic primary immunodeficiency. It is an autosomal-dominant disorder caused by heterozygous dominant-negative mutations in the transcription factor STAT3. Here, we propose clinical management guidelines for this multisystem condition based on the experience and management of these patients in the last 25+ years.

## Introduction

STAT3 hyper IgE syndrome (STAT3-HIES) is an autosomal-dominant primary immunodeficiency caused by dominant-negative variants in *STAT3*. STAT3-HIES is characterized by the triad of chronic eczema, recurrent bacterial and fungal infections, and markedly elevated serum IgE levels. Beyond immunologic dysfunction, patients frequently exhibit skeletal abnormalities, retained primary teeth, characteristic facial features, vascular malformations, and gastrointestinal complications, resulting in substantial lifelong morbidity and increased mortality (1, 2, 3, 4).

Thus far, limited guidelines exist describing concrete recommendations for the clinical management of STAT3-HIES. Therefore, this article aims to translate available evidence and clinical experience into practice-oriented management. This recommendation is based on a targeted literature review of hyper IgE syndromes and extensive long-term experience caring for >20 adult patients with STAT3-HIES at a single center. Where robust data were lacking, recommendations are extrapolated from comparable immunodeficiencies, with explicit acknowledgement of the resulting limitations. This article is structured along the clinical pathway from diagnosis and baseline assessment through to management recommendations. The goal is to support clinical immunologists and multidisciplinary specialties in delivering standardized care to patients with STAT3-HIES.

## Diagnosis and baseline evaluation

### Clinical suspicion of STAT3-HIES

Clinical suspicion of STAT3-HIES should arise in patients with a combination of chronic eczematous dermatitis, recurrent “cold” staphylococcal skin abscesses, recurrent pneumonias, and persistently elevated serum IgE levels, typically above 2,000 IU/ml. Neonates may present with heterogeneous rashes, ranging from transient papulopustular or eosinophilic folliculitis–like eruptions within the first days to weeks of life to rapidly progressive, severe eczematous dermatitis, often complicated by secondary *Staphylococcus aureus* infection (5). Other characteristic features include recurrent upper respiratory tract infections, chronic mucocutaneous candidiasis (CMC), a typical facial appearance, retained primary teeth, scoliosis, low-trauma fractures, joint hyperextensibility, lymphadenopathy, and, in a subset of patients, lymphoma (6).

Recurrent infections can typically lead to structural lung disease in the majority of adult patients with STAT3-HIES, including pneumatoceles or bronchiectasis (6, 7). The National Institutes of Health score provides a structured tool to quantify clinical suspicion and to select patients for molecular genetic testing. A score >20 points, particularly when points derive from multiple organ systems, should trigger genetic analysis (1). Importantly, the exact age at which the clinical and laboratory features (e.g., raised serum IgE levels) manifest can vary, and these features can accrue over time. This variability makes the early diagnosis of STAT3-HIES challenging in very young children. Therefore, not all features need to be present to suspect STAT3-HIES in the pediatric population.

### Genetic diagnosis

The diagnostic gold standard is molecular confirmation of pathogenic *STAT3* variants, including dominant-negative mutations (classic STAT3-HIES) and haploinsufficiency variants (8). Next-generation sequencing of genomic DNA from whole blood or peripheral blood mononuclear cells has largely replaced Sanger sequencing due to higher sensitivity and the ability to sequence multiple genes.

STAT3 dominant-negative mutations have full penetrance, however, with a very variable expressivity (2). De novo mutations have been reported to be the main cause of STAT3 dominant-negative disease; however, the relative proportion of de novo versus inherited STAT3 loss-of-function mutations may change as survival of affected individuals improves into reproductive age. STAT3 haploinsufficiency has been recently confirmed to cause autosomal-dominant STAT3-HIES in one additional family, which presented with a clinical phenotype similar to dominant-negative mutations, including recurrent skin and pulmonary infections, elevated IgE, reduced Th17 cells, and connective tissue abnormalities (9). Somatic mosaicism in *STAT3* can result in milder or intermediate clinical phenotypes and may lead to lower variant allele frequencies, requiring careful interpretation (10). Nonetheless, somatic mosaicism leading to STAT3 loss-of-function remains challenging to diagnose as it is not reliably detected using standard exome sequencing currently available in diagnostic laboratories and may be tissue-specific. Therefore, if somatic mosaicism is suspected, deep sequencing with coverage of at least 1,000× is required, and testing affected tissues is important.

In cases with variants of uncertain significance, functional testing using research assays can be performed. Primary cell-based assays include evaluation of STAT3 phosphorylation following cytokine stimulation, assessed by flow cytometry or western blot, and measurement of the IL-10–mediated, STAT3-dependent TNF-α inhibition ratio in peripheral blood mononuclear cell–derived monocytes by flow cytometry. The latter assay has been validated to discriminate between STAT3 dominant-negative, gain-of-function, and haploinsufficiency variants with high sensitivity and specificity (11). Additional approaches include cloning and expressing *STAT3* variants in cell lines (12). Assessment of STAT3 protein expression by western blot or intracellular flow cytometry can be performed; however, these methods cannot diagnose STAT3-HIES when protein expression is normal.

**Figure 1.**
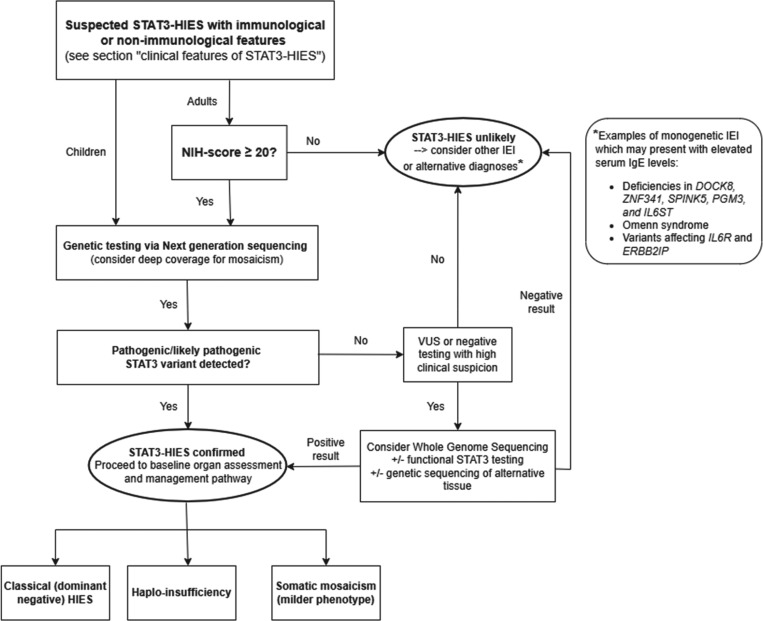
Diagnostic algorithm for STAT3-HIES.

As diagnostic techniques and knowledge of disease-associated genes continue to evolve rapidly, the storage of genomic DNA from affected individuals in whom a molecular diagnosis has not yet been confirmed is strongly recommended. Fig. 1 summarizes the diagnostic pathway of STAT3-HIES.

### Baseline organ assessment

Following diagnosis, a comprehensive baseline evaluation is essential to characterize disease extent and guide subsequent monitoring. In addition, a multidisciplinary approach should be initiated and individualized to the patient, involving specialists, for example, in immunology, pulmonology, dermatology, dentistry, orthopedics, neurology, cardiology, and infectious diseases. Suggested initial assessments include full blood count with differential, immunoglobulin quantification, including IgG subclass analysis, Th17 cells where available, and evaluation of specific antibody responses to nonconjugated-polysaccharide and protein-conjugated vaccinations.

Pulmonary function testing is recommended on a yearly basis to document the lung capacity. However, as pulmonary function testing is not very sensitive to small but progressive structural lung damage, a high-resolution computed tomography (CT) of the chest should be performed to identify pneumatoceles and bronchiectasis. Depending on the outcome of the initial baseline scan, repeat lung high-resolution CT scans may be performed in 1- to 5-year intervals.

Vascular tortuosity and aneurysms are recognized non-immunological manifestations of STAT3-HIES, most commonly affecting the coronary arteries (reported in up to 70% of patients), with less frequent involvement of cerebral, bronchial, and other arterial beds (13, 14). There is no available prevalence data for intracranial aneurysms. Nonetheless, subarachnoid hemorrhage has been rarely reported in STAT3-HIES and represents a serious and potentially fatal complication (15). In young children, initial vascular assessment should be performed using echocardiography. From the age of 10 years and onward, CT or magnetic resonance (MR) angiography of the intracranial and coronary vessels is recommended at diagnosis. Repeat imaging is advised if abnormalities are detected, guided by multidisciplinary recommendations. In patients with normal baseline imaging, surveillance cranial MR imaging (MRI) and coronary angiography every 5 years are reasonable given the progressive nature of vasculopathy. We do not recommend transthoracic echocardiography for routine detection of coronary aneurysms or ectasia, as the examination is operator-dependent and provides more limited visualization of the coronary arteries compared with CT (16).

A spinal radiograph should be performed when scoliosis is suspected. A baseline orthopedic assessment is recommended for all patients at the time of diagnosis, including screening for osteoporosis with dual-energy x-ray absorptiometry (DEXA) from the age of 5 years onward. Nonetheless, DEXA results in children should be interpreted carefully due to technical and diagnostic limitations in this age group. In children not eligible for DEXA, a pediatric endocrine evaluation should be considered. Further assessment should be considered in both adults and children if there is clinical concern for bone involvement. A baseline dental evaluation should be undertaken at the time of diagnosis (6, 17). Subsequent follow-up intervals should be individualized based on the extent of organ involvement.

## Anti-infective management

### General principles

Due to STAT3 deficiency, IL-6–driven induction of hepatic acute-phase proteins is impaired, leading to a blunted systemic inflammatory response even during significant infections. C-reactive protein levels may remain low despite clinically significant infections and inflammatory signs; e.g., skin abscesses or fractures can be minimal (6, 18). Therefore, anti-infective management focuses on early recognition and aggressive treatment of infections in the context of an attenuated inflammatory response.

Microbiological confirmation with cultures and, where appropriate, PCR from all accessible sites should be pursued systematically before or alongside therapy (19). Standard severity scores such as CRB-65 may underestimate the severity of pneumonia in STAT3-HIES, especially in patients with preexisting lung damage, therefore a low threshold for obtaining chest imaging (chest CT is favored over x-ray) is recommended.

### Acute antibacterial therapy

The most frequent infection sites in STAT3-HIES are skin, soft tissue, lungs, oral cavity, and paranasal sinuses (6, 8, 9). Common infections and pathogens in STAT3-HIES are summarized in Table 1. Acute bacterial infections should prompt immediate antibiotic therapy. Cotrimoxazole and β-lactam antibiotics such as amoxicillin/clavulanic acid are frequently used as first-line treatment because of their broad coverage and efficacy against many typical pathogens, while methicillin-resistant *Staphylococcus aureus* (MRSA) infections may require clindamycin or linezolid depending on susceptibility and local resistance patterns. The duration of therapy should reflect severity and site: 10–14 days is often adequate for uncomplicated infections, whereas severe or recurrent pneumonias may require up to 21 days of treatment.

**Table 1.** Common infections in STAT3-HIES

Infection type	Typical pathogens	Clinical features	Approximate frequency in STAT3-HIES	Recommended treatment options
Skin abscesses/cellulitis	*S. aureus* (often methicillin-susceptible *S. aureus*; sometimes MRSA)	Cold abscesses with minimal erythema/warmth; recurrent furunculosis	∼50–80% (20, 21)	• Pathogen identification and antimicrobial susceptibility testing
				• Prompt systemic antibiotics according to local resistance
				• Surgical drainage
				• Initiation of prophylactic antibiotics in patients with recurrent skin abscesses/cellulitis
Eczema-associated superinfection	*S. aureus*, β-hemolytic streptococci	Impetiginization of chronic eczema; crusted lesions	Very common (most have secondary bacterial infection of eczema at some point) (22)	• Pathogen identification and antimicrobial susceptibility testing
				• Systemic or topical antibiotics plus anti-inflammatory eczema management and antiseptic baths
				• Consider prophylactic antibiotics in patients with recurrent eczema-associated superinfection
Fungal skin infections	*Candida* spp., dermatophytes	Intertrigo, onychomycosis, tinea infections	Common, however, less systematically reported (22)	• Topical azoles or allylamides for limited infections
				• Oral antifungal agents for extensive, recalcitrant, or nail involvement
Recurrent pneumonia	*S. aureus*, *Streptococcus pneumoniae*, *Haemophilus influenzae*, respiratory viruses	Recurrent pneumonias from infancy; often progress to bronchiectasis/pneumatoceles	∼80–90% ever; >60% with ≥2 pneumonias (8, 22, 23)	• Pathogen identification and antimicrobial susceptibility testing
				• Early, culture-guided systemic antibiotics
				• Consider antibiotic prophylaxis and/or IGRT
Chronic lung infection	*S. aureus*, *P. aeruginosa*, *Aspergillus fumigatus*	Structural lung disease (bronchiectasis, pneumatoceles); chronic productive cough	Structural changes are present in ∼80–95% adults; chronic bacterial/fungal colonization is common (22)	• Prolonged, culture-guided systemic or inhaled antimicrobials when appropriate
				• Intensive airway-clearance strategies
				• Multidisciplinary care with involvement of pulmonology
				• Consider referral to surgery for complications, e.g., pneumatoceles
CMC (oral, genital, nails)	*Candida albicans* and other *Candida* spp.	Recurrent oral thrush, angular cheilitis, vulvovaginal candidiasis, onychomycosis	∼50–85% across cohorts (21, 22)	• Pathogen identification and antimicrobial susceptibility testing
				• Topical azole or nystatin therapy for limited infections
				• Systemic fluconazole (or alternate azole based on species and resistance) for refractory infection
				• Consider long-term prophylaxis with systemic antifungal agent (e.g., fluconazole)
Severe disseminated infections (sepsis, deep abscesses)	Mainly *S. aureus*; occasionally gram-negatives	Invasive infections often from skin/lung focus	Less common; however, major cause of morbidity/mortality (22, 24)	• Urgent medical treatment, including broad-spectrum intravenous antibiotics with prompt de-escalation according to cultures and source
				• Aggressive source control (e.g., drainage of abscesses)
				• Review long-term prophylaxis
				• Consider IGRT
				• Consideration of HSCT in selected patients with severe, refractory infectious complications

### Long-term antibacterial prophylaxis

Due to the high risk of recurrent, chronic, and potentially life-threatening infections, many patients with STAT3-HIES benefit from long-term antibacterial prophylaxis. The regimen selection should be individualized based on age, infection history, lung status, and local resistance patterns. Table 2 outlines common antimicrobial prophylaxis regimes.

**Table 2.** Recommended antibiotic prophylaxis in STAT3-HIES

Type of infection	Antibiotic	Adult dose^a^	Pediatric dose^a^
Recurrent skin infections (≥3 cold abscesses/year) or recurrent infections with MRSA colonization	Cotrimoxazole (Trimethoprim/Sulfamethoxazol)	960 mg daily	5 mg/kg/day trimethoprim component, given once daily or divided twice daily (max 160 mg trimethoprim/day)
Recurrent sinopulomonary infections requiring antimicrobial treatment (≥2 episodes/year)	Azithromycin	250 mg three times weekly (e.g., Monday/Wednesday/Friday)	5–10 mg/kg (max. 250 mg) three times weekly (e.g., Monday/Wednesday/Friday)
Second-line for recurrent skin and sinopulmonary infections	Amoxicillin-clavulanate	500 mg/125 mg twice daily	20–25 mg/kg/day amoxicillin component, divided twice daily (use high-dose 40–45 mg/kg/day in selected high-risk patients)

a Final choice of drugs, doses, and durations should be individualized according to local infectious diseases guidance, age, weight, renal/hepatic function, and susceptibility patterns.

### *Pseudomonas* eradication

Early eradication of *Pseudomonas aeruginosa* from lower airways is strongly recommended (evidence adapted from bronchiectasis and cystic fibrosis guidelines). Therapy should be initiated within 4 wk of first microbial isolation to prevent chronic infection. Inhaled eradication therapy is recommended as a first-line treatment approach (22, 25, 26). If inhalation is not feasible, an intravenous regimen with antipseudomonal β-lactams should be considered (Table 3). Negative sputum cultures at 1, 3, 6, and 12 mo after treatment confirm success of the eradication therapy. Lifelong prophylaxis (for example, with azithromycin/cotrimoxazole) is often required despite eradication, due to the high prevalence of structural lung disease.

**Table 3.** Antimicrobial strategies for *P. aeruginosa* eradication in STAT3-HIES

Regimen^a^	Duration	Notes
Inhaled tobramycin	4 wk	First-line if inhalation feasible
Ciprofloxacin orally 3 wk + inhaled colistin 3 mo	3 mo	Combination superior to systemic alone
Intravenous antipseudomonal β-lactam + aminoglycoside	14 days	Inhalation not feasible

a Final choice of drugs, doses, and durations should follow local cystic fibrosis/bronchiectasis protocols and should be individualized to age, weight, renal/hepatic function, and susceptibility testing.

### Antifungal therapy

Individuals with STAT3-HIES are more susceptible to fungal infections overall; therefore, clinicians should maintain a high index of suspicion for fungal infection. Cutaneous fungal infections are particularly common. CMC, involving the oral, axillary, inguinal, and nail regions, is a frequent manifestation in STAT3-HIES and often necessitates combined systemic and topical antifungal therapy. Systemic fluconazole 100–200 mg once daily in adults or 3–6 mg/kg once daily in children (the exact dose should be individualized based on age, ideally in consultation with pediatric infectious diseases) is effective in preventing recurrent CMC, while topical agents such as nystatin or clotrimazole and antiseptic baths support local disease control. Other fungal skin infections observed more frequently in individuals with STAT3-HIES include primary invasive cutaneous fusariosis and dermatophytosis (27). Importantly, adequate control of eczema, for example, through treatment with an anti-IL-4Rα monoclonal antibody, significantly reduces the risk of secondary skin infections (28).

Individuals with STAT3-HIES and structural lung disease are at increased risk of pulmonary mold infections, including secondary infection of pneumatoceles, particularly with *Aspergillus* species. In patients with prior *Aspergillus*-positive bronchoalveolar lavage or culture, structural lung abnormalities, or recurrent pneumonias despite antibacterial prophylaxis, antifungal prophylaxis against *Aspergillus* species should be considered, guided by imaging findings, antimicrobial resistance patterns, and potential drug–drug interactions. Itraconazole 200 mg once to twice daily in adults and itraconazole suspension 5 mg/kg once daily in children are the preferred first-line agent. Posaconazole 300 mg delayed-release tablets once daily may be used in cases of itraconazole intolerance or for breakthrough *Aspergillus* infections despite itraconazole therapy. Therapeutic drug monitoring is important for patients receiving itraconazole or posaconazole prophylaxis due to variable absorption and the need to reach adequate trough concentrations.

In addition to cutaneous and pulmonary fungal disease, patients with STAT3-HIES are at increased risk of opportunistic fungal infections, including *Pneumocystis jirovecii* pneumonia, disseminated histoplasmosis, and cryptococcosis, which should be treated in accordance with current clinical guidelines (8).

## Immunoglobulin replacement therapy (IGRT)

Clinical experience suggests that IGRT can reduce the frequency and severity of infections and the progression of structural lung disease in selected patients with STAT3-HIES (25). IGRT should be considered in patients with STAT3-HIES with one of the following: recurrent severe infections despite optimized anti-infective prophylaxis, low total IgG levels, selective IgG2 deficiency, impaired specific antibody responses, and/or established bronchiectasis or other chronic lung changes. IGRT can be administered intravenously at ∼400 mg/kg every 3–4 wk or subcutaneously at ∼100 mg/kg per week, with the route chosen according to patient preference, venous access, and logistical factors.

Adverse effects of IGRT include local reactions at subcutaneous infusion sites and systemic symptoms such as headache, back pain, fever, and nausea with intravenous infusions. Severe complications such as anaphylaxis, thromboembolic events, renal impairment, and hemolytic anemia are rare, however, require appropriate monitoring and counseling. Systemic adverse effects in subcutaneous replacement are rare.

## Organ-specific management

### Skin involvement

#### Standard topical therapies

Severe, chronic eczema is one of the most prominent and disabling manifestations of STAT3-HIES and substantially increases the risk of cutaneous infections. Baseline management should follow established dermatologic guidelines for eczema treatment. Regular use of emollients, topical corticosteroids, or topical calcineurin inhibitors, together with episodic treatment using topical antibiotics or antifungals when indicated, constitutes the foundation of maintenance therapy. Weekly or more frequent decolonization and disinfection of the skin using bleach baths (e.g., 1/2 cup of sodium hypochlorite in a bathtub for 15 min, three times per week), salt baths or antiseptic washes (e.g., chlorhexidine-containing solutions), and the use of intranasal mupirocin can significantly reduce the frequency of staphylococcal skin infections.

#### Targeted therapies

Monoclonal antibodies targeting key cytokines involved in type 2 inflammation represent a pathogenetically rational therapeutic strategy for severe eczema and have revolutionized the management of eczema in STAT3-HIES. Dupilumab, an anti–IL-4Rα monoclonal antibody that inhibits IL-4 and IL-13 signaling, is currently the most commonly used biologic agent in STAT3-HIES, supported by an established safety profile in case reports involving patients with immunodeficiency and the largest body of clinical experience to date. Case reports have demonstrated marked efficacy of dupilumab in STAT3-HIES, with complete resolution of eczema in most cases and no reported opportunistic infections despite the underlying immunodeficiency (21, 28, 29). Dupilumab dosing follows the recommendations established for the treatment of atopic dermatitis. For pediatric patients, dosing is weight-based and varies by age group. The standard regimen for adults consists of a 600 mg loading dose followed by 300 mg administered subcutaneously every 2 wk, with individual dose adjustments based on clinical response and tolerability. Given the underlying immunodeficiency, close monitoring for infections and atypical adverse events is mandatory but have only rarely been reported. In rare cases of uveitis, the drug does not necessarily need to be discontinued, as this side effect may wean with time.

#### Further supportive measures

Abscesses require incision and drainage with sampling for microbiological culture. The primary closure of the cavity should be avoided to reduce recurrence risk. The education of patients and caregivers on early recognition of skin infections and prompt presentation is crucial in preventing severe skin-related complications.

### Pulmonary involvement

High-resolution CT chest (preferably using a non-contrast, low-dose protocol) is essential at diagnosis to characterize structural lung disease, which is common (80–95% of patients) in STAT3-HIES (22, 26). Annual follow-up with spirometry and a 6-min walk test is recommended for patients with pneumatoceles or bronchiectasis to monitor for progressive respiratory failure (8, 19). Surveillance sputum microbiology every 6–12 mo is recommended in patients with structural lung disease.

All patients with lung involvement should receive training in chest physiotherapy. Regular airway clearance should be performed at least twice daily, including the use of airway clearance devices and inhalation of hypertonic saline. Nonetheless, airway clearance techniques need to be carefully selected and individualized in view of the increased risk of rib fractures in individuals with STAT3-HIES. Additionally, hypertonic saline should be used in caution in patients with active or recent significant hemoptysis, as this could worsen bleeding. Regular sports and structured exercise training can significantly improve airway clearance and exercise tolerance in bronchiectasis, with evidence suggesting reduced exacerbation frequency (30). Lobectomy may rarely be required in patients with large, chronically infected pneumatoceles. However, surgical lung interventions should be considered only as a last resort following thorough multidisciplinary evaluation, given the risk of impaired expansion of the remaining lung and additional complications.

### Vascular abnormalities

Vascular anomalies, including intracranial aneurysms and coronary artery ectasias, are common in STAT3-HIES, having been identified mainly through CT and MRI in research cohorts (25, 31). These vascular abnormalities may progress in the absence of clinical symptoms, yet they can pose a substantial risk of intracranial hemorrhage or myocardial ischemia. Screening with CT or MR angiography of intracranial and coronary vessels should therefore be considered for all STAT3-HIES patients. When abnormalities are present on initial imaging, individualized follow-up imaging intervals are recommended, based on lesion size, progression, and multidisciplinary cardiovascular assessment. If baseline CT or MR angiography of the intracranial and coronary vessels shows no abnormalities, repeat MRI (preferably with MR angiography) every 5 years may be considered, as vasculopathy can develop later in the disease course. Echocardiography should be considered in patients with severe pulmonary disease to evaluate for pulmonary hypertension. Importantly, individuals with STAT3-HIES should undergo regular cardiovascular monitoring from early adulthood, including lipid profiling and blood pressure assessment, to facilitate early detection and management of modifiable cardiovascular risk factors. Smoking and vaping should be strongly discouraged, and cessation services should be offered where available.

### Lymphoproliferation

Annual clinical evaluation for lymphoproliferation, including history, physical examination, and blood tests such as lactate dehydrogenase and lysis parameters, is recommended due to the increased risk of lymphoma. Symptom-driven imaging should be performed as needed, and routine imaging (e.g., annual abdominal ultrasound) may be considered in certain cases. Suspicious lymph nodes should be further evaluated with imaging and, where appropriate, excision in toto.

### Musculoskeletal system

Osteoporosis and bone fragility are common in STAT3-HIES, with minimal trauma fractures (often multiple) occurring in 50% of patients. Reduced bone mineral density (BMD) is identified in the majority of individuals with STAT3-HIES. The increased risk of osteoporosis in STAT3-HIES results from STAT3 deficiency leading to increased osteoclastogenesis through impaired IL-6/STAT3-mediated inhibition of RANKL signaling (32). Notably, radial BMD (not femoral or spinal) correlates with fracture risk, reflecting the unique skeletal pathology (33).

Therefore, we recommend performing a BMD assessment in all individuals with STAT3-HIES at the time of diagnosis, including radial BMD measurement. Repeat BMD testing should be considered every 1–2 years if bone density is reduced or risk factors change, consistent with guidelines for high-risk populations. Pharmacologic treatment (e.g., with bisphosphonates) as per international osteoporosis guidelines should be considered for patients with a high fracture risk (34).

Due to diminished inflammatory responses in STAT3-HIES, fractures may present with less pain, swelling, or other typical signs of injury. Therefore, patients and families should be educated about fracture risk, the potential for atypical or subtle fracture presentations, and fall prevention measures. Lifestyle and supportive measures to maintain bone health, such as calcium and vitamin D supplementation, regular weight-bearing exercise, and fall prevention strategies, are recommended for all adult individuals with STAT3-HIES, regardless of whether osteoporosis has developed.

Scoliosis is common in patients with STAT3-HIES and should be identified early through clinical examination, with referral for orthopedic evaluation and spinal radiographs when kyphoscoliosis is suspected. Furthermore, severe scoliosis, particularly with large thoracic curves, can lead to a reduction in lung volumes and restrictive ventilatory dysfunction, which may contribute to respiratory symptoms or complications. Regular physiotherapy slows progression of scoliosis and bracing is indicated for Cobb angle 20–40° in growing patients. Surgical correction is reserved for progressive curves >45–50° (22, 35).

Joint hyperextensibility affects at least 33% of patients, predisposing to early degenerative joint disease. Therefore, tailored physiotherapy emphasizing proprioceptive training and closed-chain exercises may be required to stabilize hypermobile joints (22).

### Dental care

**Figure 2.**
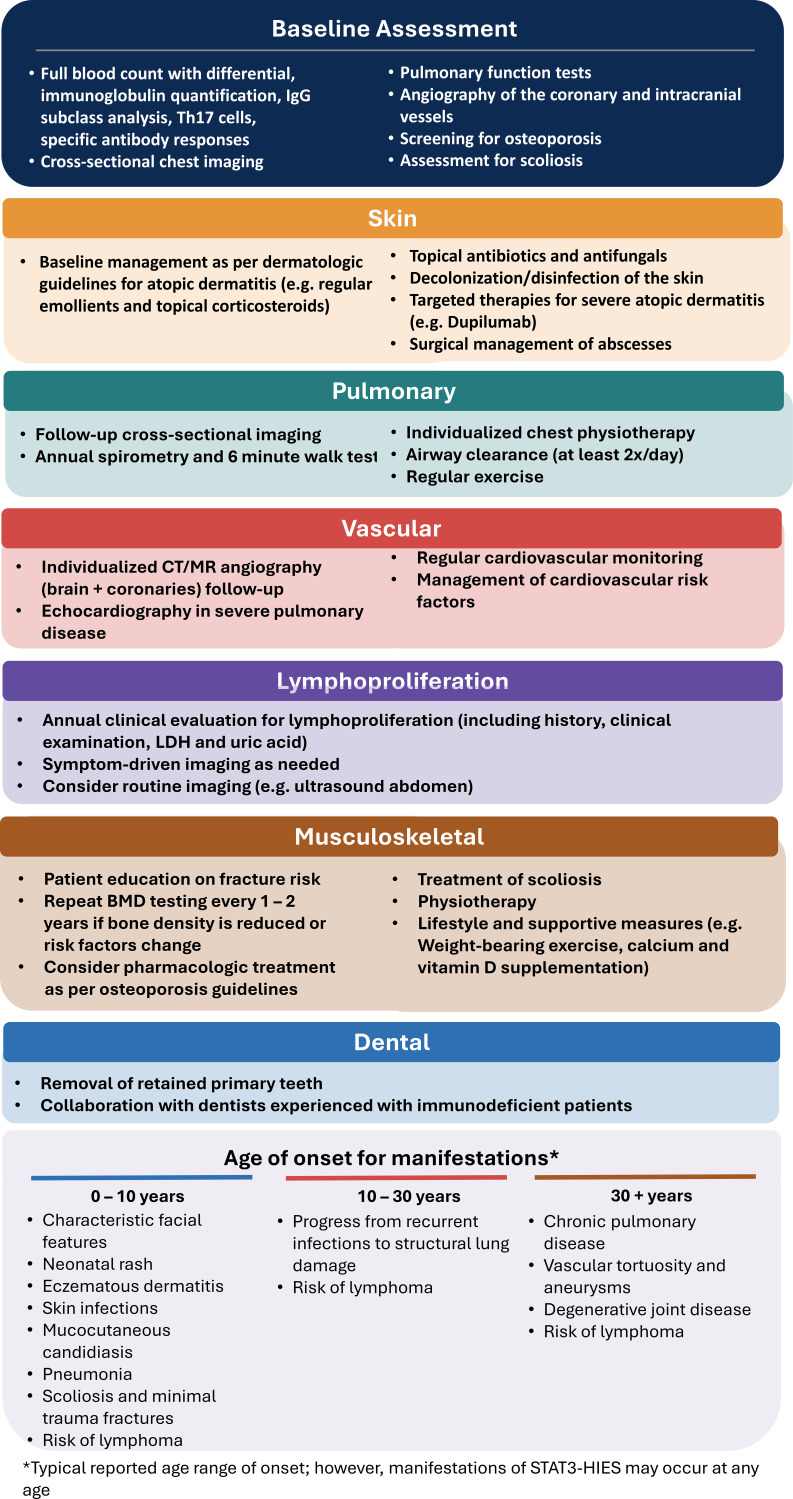
**Organ-specific** **manifestations of STAT3-HIES.**

Retained primary teeth are characteristic and should be removed at the expected time of physiological exfoliation to avoid malocclusion and other complications (17). In patients receiving bisphosphonates, the risk complications following dental extraction, such as jaw bone necrosis, is generally low for primary teeth but higher for permanent teeth. Dental extractions should ideally be timed to allow spacing from bisphosphonate administration. Management must be individualized and planned in close collaboration with experienced dentists familiar with immunodeficient patients. Fig. 2 summarizes the organ-specific management of STAT3-HIES.

## Family planning and genetic counseling

Molecular genetic testing of at-risk family members (parents, siblings, and offspring) of patients with STAT3 loss-of-function mutations is recommended once a patient is diagnosed with STAT3-HIES to allow for early diagnosis and prompt initiation of treatment and preventive measures. The optimal time to discuss genetic risk is before pregnancy planning. As STAT3-HIES is an autosomal-dominant condition, genetic counseling should include clear information on the 50% transmission risk to each offspring. The option of preimplantation genetic testing in conjunction with in vitro fertilization should be discussed in accordance with each country’s specific guidelines and regulatory framework. Prenatal diagnostic options, including chorionic villus sampling and amniocentesis, may be considered where available.

## Considerations for women of childbearing age

During menses, 50% of patients report worsening of eczema and 13% report worsening of pulmonary symptoms, such as increased shortness of breath and sputum production (36). Therefore, intensified skin care may be warranted (see section on skin involvement). Additionally, pulmonary monitoring with peak flow measurement or pulse oximetry may be considered during menses in patients with baseline lung disease.

CYP3A4-mediated drug interactions between azole antifungals and estrogen-containing oral contraceptives may reduce combined oral contraceptive efficacy and increase unplanned pregnancy risk. Importantly, estrogen-containing methods are also associated with thromboembolic risk, which is already elevated in STAT3-HIES due to the high prevalence of middle-sized artery vasculopathy. Progestin-only preparations are therefore recommended, with progestin-releasing intrauterine devices (e.g., levonorgestrel intrauterine system) being first-line based on a cohort study of women with STAT3-HIES (36).

## Obstetric and gynecological care

### Gynecological infections

Gynecological infections are frequent and often severe in women with STAT3-HIES. In a cohort of 42 women with STAT3-HIES, 30 (71%) reported recurrent perineal or breast abscesses, including 19 with breast, 15 with labio-perineal, and 5 with vaginal abscesses. Recurrent vulvovaginal candidiasis is also very common in female patients with STAT3-HIES, and many women require chronic or intermittent azole suppression therapy. Additionally, non-*Candida *vaginitis and bacterial vaginosis are reported at an increased frequency in women with STAT3-HIES (37). These observations underscore the need for close gynecological follow-up and individualized antimicrobial and antifungal strategies in this population.

### Pregnancy

Pregnancy in women with STAT3-HIES requires careful preconception counseling and multidisciplinary management. Antimicrobial prophylaxis during pregnancy should be adjusted to non-teratogenic agents (for example, penicillin, cephalosporins, or macrolides) and individualized to the patient’s pulmonary status, risk status, infection history, and antimicrobial resistance patterns, as discontinuation of antimicrobial prophylaxis may increase the risk of severe perinatal infections (8). Antifungal prophylaxis with azoles should be avoided in pregnancy. IGRT needs to be increased by ∼50% in the third trimester to provide sufficient nest protection to the unborn child.

Reports of pregnancy in individuals with STAT3-HIES include uncomplicated pregnancies as well as cases with postpartum deterioration of lung disease, potentially related to reduced antimicrobial therapy, delayed radiologic diagnosis, and impaired pulmonary clearance during pregnancy (22). Therefore, female patients with STAT3-HIES who are planning pregnancy should be counseled regarding the associated risks, particularly those with significant pulmonary restriction, severe scoliosis, or other major complications of STAT3-HIES (36).

## Hematopoietic stem cell transplantation (HSCT)

HSCT in adults is associated with considerable risks, including graft-versus-host disease, severe infections, organ failure, and neurological complications, and is therefore not routinely recommended in adult patients with STAT3-HIES. However, HSCT should be considered early on in childhood, particularly in the presence of a suitable donor (matched sibling donor or matched unrelated donor), as (1) organ damage following recurrent infections, especially the lungs and joints, become worse over time; (2) the total “infectious load” is less in infants than in adults; (3) HSCT outcomes are generally considerably better in infants than in adults. HSCT should only be undertaken in experienced centers with close long-term follow-up. Importantly, HSCT will correct the immunodeficiency and improve infection control; however, as STAT3-HIES also affects non-hematopoietic cells such as cells of the connective tissue, it may not necessarily ameliorate vascular or skeletal abnormalities (24, 31, 38). Nonetheless, prospective evidence is still lacking.

## Physiotherapy, psychosocial support, and nutrition

Scoliosis, hyperextensible joints, and recurrent joint infections make general physiotherapy indispensable to increase muscle strength and maintain joint stability and mobility. Moreover, lung-focused chest physiotherapy is a central cornerstone of maintaining lung health and is strongly recommended to maintain or improve lung function.

Managing a chronic, multisystem disease imposes a considerable psychological burden; therefore, access to psychological counseling, chronic disease management programs, and patient support groups can provide crucial help in coping with emotional challenges and improving quality of life. In addition, opportunities for patient-to-patient networking, such as patient meetings or educational events, or dedicated patient days are highly valuable, as they encourage peer support, shared experiences, and patient empowerment.

In case laboratory testing reveals deficiencies, patients with STAT3-HIES may benefit from targeted nutritional supplementation, including vitamin D to support bone health and immune function, vitamin A for skin and mucosal integrity, and zinc and vitamin C to support immune responses. However, regular monitoring is required to avoid vitamin overdosing.

## Vaccinations

In general, vaccinations should be administered according to standard immunization schedules. In patients receiving IGRT, booster vaccinations against certain toxins or bacterial pathogens are superfluous due to passive antibody protection. Nevertheless, immunization against selected pathogens in patients receiving IGRT remains particularly important, including annual influenza vaccination, SARS-CoV-2 vaccination, recombinant herpes zoster vaccination, human papillomavirus vaccination, and tick-borne encephalitis vaccination in endemic areas.

## Prognosis

The prognosis of STAT3-HIES has significantly improved over recent decades, with most individuals currently surviving into or beyond the fifth decade of life. Nevertheless, patients should be referred to an experienced center for longitudinal care. Pulmonary complications represent the most significant source of morbidity. The majority of individuals with STAT3-HIES experience pneumonia, and recurrent infections can damage lung parenchyma, leading to bronchiectasis, pneumatoceles, and secondary chronic infections, such as aspergillosis. The primary causes of death are gram-negative or filamentous fungal pneumonias, which can invade damaged lung tissue, involve the pulmonary vasculature causing hemoptysis, or disseminate to multiple organs. The use of antimicrobial prophylaxis and IGRT has dramatically reduced pneumonia recurrence (25). HSCT is a treatment option if a suitable donor is found and effectively corrects the immunological defects and can stabilize severe lung disease. However, the impact of HSCT on nonimmune manifestations, such as skeletal abnormalities, appears limited (24). Additional mortality risks include myocardial infarction due to coronary artery aneurysms and subarachnoid hemorrhage from intracranial aneurysms. Lymphomas, primarily non-Hodgkin type, also occur at increased frequency, whereby standard chemotherapy has been successful in reported cases (39, 40, 41).

## Discussion

This article integrates long-term clinical experience with the available published evidence into a structured, management-oriented framework for the care of patients with STAT3-HIES. It provides diagnostic tools and treatment schemes with practical algorithms, including anti-infective protocols with specific treatment recommendations and dosing regimens, long-term prophylaxis strategies, and rational indications for IGRT. Comprehensive management and monitoring recommendations for complications associated with STAT3-HIES are included.

An important limitation of this article is that most recommendations are based on observational data and expert opinion. Prospective studies are needed to better define the outcomes of current clinical management strategies in patients with STAT3-HIES, especially with regard to the very important question whether or not HSCT in childhood leads to an improved quality of life and survival in adulthood. In keeping with recent developments in the treatment of severe atopic dermatitis, future studies are required to establish the safety and efficacy of alternative targeted treatments, such as biologic agents blocking IL-13 or IL-31R (tralokinumab, lebrikizumab, and nemolizumab) in the treatment of skin manifestations of STAT3-HIES. Additionally, further research is required to define strategies to address non-immunologic complications of STAT3-HIES.

In view of the rarity of this condition, participation in international disease registries and collaborative research initiatives is essential to enable adequately powered cohort studies and support the development of evidence-based management recommendations. An international Delphi consensus process should be initiated to address this gap and to develop an international treatment guideline for the management of STAT3-HIES.
